# Management of Thromboembolic Disease in Patients with Primary and Metastatic Brain Tumors

**DOI:** 10.1007/s11864-023-01116-w

**Published:** 2023-07-06

**Authors:** Ryan R. Woods, Glenn J. Lesser

**Affiliations:** Wake Forest School of Medicine, Medical Center Blvd, Winston-Salem, NC 27157 USA

**Keywords:** VTE, Venous Thromboembolism, Pulmonary embolism, Deep venous thrombosis, Brain tumor anticoagulation, Glioblastoma multiforme, Thrombosis, Anticoagulation

## Abstract

Patients with primary brain tumors are at a substantially elevated risk of venous thromboembolism (VTE) compared to other disease states or other forms of malignancy. Deep venous thrombosis (DVT) and pulmonary embolism (PE), often complicate the care of patients with primary brain tumors, and treatment may pose specific unique risks and considerations for management. This paper critically reviews the relevant literature and the most common treatment options in addition to a discussion regarding the relative risk considerations for neurooncology patients facing thromboembolic disease.

## Introduction

Cancer has been long recognized as an independent and significant risk factor for venous thromboembolism (VTE) [1]. Patients with primary brain tumors are a particularly vulnerable subgroup, likely due to an underlying hypercoagulability associated with brain tumors in addition to risks associated with surgery or paresis [2]. The presence of IDH1 mutations has been recently described as a risk factor [3]. While most patients derive clear benefit from anticoagulation, patients with primary brain tumors or metastatic disease to brain, may be at a real or perceived heightened risk of bleeding that often muddles clinical decision making.

Complicating the issue further is the fact that new anticoagulant clinical trials often initially exclude cancer patients potentially out of concern for early mortality (and censoring which compromises trial endpoints), drug-drug interactions, or safety concerns (e.g. development of treatment-related thrombocytopenia) [4–6]. Patient-related factors may also be present that can increase the mortality risk of any one event, such as frailty, preexisting pulmonary hypertension or other cardiopulmonary disease [7]. The cumulative result is that most data in regards to anticoagulation is first driven by the experience in non-cancer settings which is then abstracted to the cancer patient. Ideally, this experience is then backed up with subsequent trials and real-world data.

A key tenet of good anticoagulant management is based upon a fairly weighted and ongoing risk assessment performed by the clinician. This paper will first review the data in regard to these issues from a high-level perspective, and then focus on data available related to the experience in patients with primary and metastatic brain tumors. This will be followed by a review of therapeutic options currently available to the practicing clinician, but with emphasis on the newer data in support of more recently adopted oral anticoagulants.

### Risks of acute VTE

Untreated acute VTE poses risks of mortality and morbidity, perhaps by pulmonary embolism and subsequent cardiovascular collapse. In some studies, mortality attributed to any single untreated symptomatic pulmonary embolism approaches 30%, usually via a recurrent or cumulative embolic event [8]. Isolated calf Deep Venous Thrombosis (DVT) appears to be associated with a significantly lower risk compared to more central and proximal DVT [9, 10]. Therefore, in addition to patient-related risk factors, the practicing clinician is encouraged to consider the risk associated with a particular thrombosis, including the relative onset, location, proximity, and extent. The large central acute PE and the isolated distal calf DVT should probably be considered for anticoagulation with different ferocity.

### Benefit and risks of anticoagulation

Anticoagulants have been long recognized as effective initial therapy for VTE as well as for secondary prevention in long-term settings. Highlighting the need for rapid intervention, in one study conducted at the Mayo clinic, patients diagnosed with PE who start anticoagulation while still in the ED were noted to have significantly less mortality compared to patients who start anticoagulation following admission to hospital [11]. In this study, patients who received anticoagulation in the ED demonstrated a dramatic difference in in-hospital (1.4% versus 6.7%, *p* = 0.009) and 30-day mortality (4.4% vs 15.3%, *p* < 0.001), compared to patients starting anticoagulation outside of the ED. Beyond the immediate setting, it is clear that the risks from VTE are substantially lower following 3 months of anticoagulant, with some debate over the relative benefit of 6 months of primary treatment [12]. After 3–6 months of anticoagulation, additional risk-assessment is helpful to determine the potential benefit of long-term therapy, as secondary prophylaxis [13].

Anticoagulants inhibit secondary hemostasis, and are therefore thought to not directly trigger spontaneous bleeding, but may compromise hemostatic mechanisms that may have otherwise been resolved without coming to clinical attention, including cerebral microbleeding [14]. In regards to primary brain tumors, risk of both spontaneous bleeding as well as risk of bleeding with anticoagulation exposure appears to trend with grade. GBM appears to incur a risk of spontaneous intracranial hemorrhage (ICH) of approximately 5% [15]. A specific setting to note is in the primary pituitary tumor, where apoplexy is reported to be high as 14% even in the absence of anticoagulation [16]. Concurrent thrombocytopenia, perhaps secondary to myelosuppressive chemotherapy, increases the risk of bleeding but the specific correlation is loosely defined in this setting. The use of VEGF inhibitors have been associated with an increased risk of bleeding complications. In the AVAglio trial, the addition of bevacizumab was associated with a risk of CNS bleeding of 3.3%, compared to 2.0% in patients receiving temozolomide and radiotherapy without bevacizumab. This difference was not statistically significant. Non-CNS bleeding was significant however, with 37.1% of patients receiving bevacizumab experiencing at least a grade 1 bleeding event versus 19.6% of patients receiving the control regimen (*p* < 0.001) [17]. The similar RTOG 0825 trial reported no statistical difference in CNS bleeding, but did not report similar non-CNS bleeding [18].

### Anticoagulant options

This review will attempt to summarize the significant data in support of the major classes of anticoagulants in common practice to better clarify the risks and benefits as they apply to the neuro-oncology patient.

#### Vitamin K antagonists

Vitamin K antagonists (VKA) such as warfarin represent the oldest class of anticoagulants and are effective at reducing the risks of thrombosis. Warfarin is encountered as a control arm in more recent data and does reduce the risk of recurrence or VTE-related death [19]. However, its use has been largely supplanted in the cancer patient for multiple reasons including good data to suggest higher rates of recurrent thrombosis, and more bleeding when compared to other oral agents [4, 5]. In addition, VKA has high potential for drug-drug interactions (particularly via CYP29C) or diet-related factors, which compromise their risk profile. Still, VKA dosed to therapeutic INR 2–3, remains a potential option in the highly selected patient (i.e., for one who convincingly fails primary and secondary alternatives, declines injections, and passes a risk/benefit assessment to warrant anticoagulant). There are no large studies focused on the neuroncology patient from which to draw firm conclusions, and therefore this agent should be relegated as a reserve option.

#### Low molecular weight heparins (LMWH)

Until recently, low molecular weight heparins had largely been accepted as the preferred anticoagulant for cancer patients with acute VTE owing to data which suggested better efficacy and lower risks compared to other available agents. A landmark study that established this as a standard was the CLOT trial, which was reported in 2003 [19]. This multicenter prospective interventional trial randomized 676 cancer patients with acute proximal DVT (including popliteal vein or further cephalad) and radiologically confirmed PE, to receive standard-dose low molecular weight heparin (dalteparin) versus VKA. A total of 27 patients had primary brain tumors (4.5%). 27 of 336 (8%) patients who received LMWH had recurrent venous thromboembolism within 6 months compared with 53 of 336 patients (15.7%) in the warfarin group (hazard ratio, 0.48; *P* = 0.002). Other relevant trials that offered additional support of this approach included the ONCENOX and CATCH trials [20, 21]. With these and other data, the American Society of Clinical Oncology first published guidelines on this topic in 2007 with updates in 2013 and 2015, establishing LMWH as the preferred agent for initial and subsequent treatment for cancer patients with VTE [22].

There have been subsequent retrospective analyses investigating the rates of bleeding within the neuroncology patient population following anticoagulation. These data suggest that the rate of ICH of high-grade primary brain tumors when treated with standard dose LMWH appears to be between 4.7 and 14.7% [22–24]. Chai-Adisaksopha et al. reported a retrospective total of 364 patients with cancer-associated VTE [23]. In this report, 67 patients had primary brain tumors and a total of 115 patients had metastatic disease to brain. LMWH was effective at reducing risks and sequelae of VTE, but came at a cost of increased intracranial bleeding witnessed in patients with brain tumors versus other malignancies (4.9% vs. 0%, *p* = 0.04). Additional data is variable and includes smaller numbers of patients. Mantia et al. reported on 50 patients with high grade glioma treated with enoxaparin and identified a significantly higher rate of ICH compared to those who received no anticoagulation (14.7% vs. 2.5% HR 3.37, 95% CI 1.02–11.14) [22]. The rate of intracranial bleeding was not statistically different between metastatic and primary CNS tumors (5.97% vs 3.48%, *p* = 0.48). Yust-Kats reported on the role of the Khorana scale to evaluate the risk of VTE and offered some data of bleeding risk in GBM patients [24]. In this study, 64 patients developed VTE of 440 patients with GBM over the course of treatment. Of the 36 patients who received anticoagulation, 2 developed ICH (5.5%).

#### Direct oral anticoagulants (DOAC)

The development of effective oral agents that do not require monitoring adjustments has significantly changed the approach to anticoagulation. Commonly encountered agents in this space include the inhibitors of Factor Xa (rivaroxaban, apixaban, and recently edoxaban) and the direct thrombin inhibitor, dabigatran.

##### Apixaban

The AMPLIFY trials led to the approval of apixaban as primary therapy for acute VTE. A total of 5400 patients were randomized to conventional therapy (LMWH followed by warfarin to INR 2–3) or apixaban, 10 mg twice daily for 7 days, followed by 5 mg twice daily [4]. The trial demonstrated noninferiority in regards to its primary endpoint of recurrent VTE or VTE-related death; 2.3% in the apixaban group compared with 2.7% in the conventional-therapy group (RR, 0.84; 95% CI 0.60–1.18). Apixaban was found to be superior in regards to major bleeding (0.6% in apixaban group compared to 1.8% in control arm (RR, 0.31; 95% CI, 0.17 to 0.55; *P* < 0.001). AMPLIFY did exclude cancer patients, including those with primary brain tumors.

##### Rivaroxaban

The development of rivaroxaban parallels that of apixaban, with similar trial design and experience. FDA approval followed the reporting of the phase 3 experience in both DVT and PE settings and a preplanned pooled analysis [5, 25, 26]. In the pooled analysis, a total of 8282 patients received rivaroxaban 15 mg twice-daily for 21 days, followed by 20 mg once-daily compared to standard-therapy (enoxaparin 1.0 mg/kg twice-daily followed by VKA) [26]. The primary efficacy outcome, the rate of symptomatic recurrent VTE, was 2.1% in the rivaroxaban group compared to 1.8% for control (HR 1.12; 95% CI 0.75–1.68). There likewise was no significant difference in composite bleeding, but the data did suggest a reduction in major bleeding of 1.1% compared to 2.2% for the LMWH arm (HR 0.49; 95% CI 0.31–0.79; *P* = 0.003 for superiority). The trial contained a small cancer subgroup. A total of 430 patients (5.1%) of subjects were deemed to have “active cancer.”

##### Edoxaban

Edoxaban was approved following outcomes largely similar to prior DOAC experiences. This agent has the practical drawback that it is not approved for use in the first 5 days of treatment and there are concerns regarding accelerated clearance in patients with an excellent glomerular filtration rate (> 95 ml/min) [27]. This agent is notable having been the subject of the first randomized, prospective trial of a DOAC conducted solely in cancer patients [28•]. The Hokusai-VTE cancer trial prospectively randomized 1050 cancer patients to receive open-label edoxaban 60 mg once daily versus standard-dose LMWH (dalteparin) [28•]. In the trial, edoxaban was again found to be non-inferior in regard to recurrent VTE (HR 0.97; 95% CI 0.70–1.36, *P* = 0.006). Major bleeding was similar between the two arms (6.9% edoxaban vs 4.0% in control, HR 1.77, 95% CI 0.71–1.36). There was an increased rate of major bleeding in patients with gastrointestinal cancer (13.2% vs 2.1%) that was not evident in other tumor types. A total of 2 of 522 patients in the edoxaban group developed intracranial hemorrhage compared to 4 of 524 receiving control, suggesting no significant increased risk of ICH attributed to the agent. Patients with primary brain tumor and metastatic tumors to brain were included, but not well enumerated (108 classified within an “other solid tumors” category). There were also patients represented who received bevacuzimab therapy prior to anticoagulant treatment (15.8% and 16.1% between edoxaban and LMWH groups) as well as a smaller group who received anti-VEGF therapy randomization (2.5% and 3.2% respectively). This trial occurred well after LMWH gained wide acceptance as a standard therapy in patients with cancer. As such, the control arm did not include use of VKA in contrast to the general DOAC studies, which usually compared the new oral option against the conventional oral comparator, VKA. Therefore, this trial arguably offers a more relevant control baseline as opposed to the other DOAC trials when considering cancer patients.

##### Dabigatran

The direct-thrombin inhibitor dabigatran was first approved for stroke prevention in atrial fibrillation, with subsequent label expansion in April 2014 to include DVT and PE following the RE-COVER trials [29]. The primary endpoint of recurrent VTE or VTE-related death at 6 months was 2.4% for dabigatran and 2.1% for the warfarin control (HR 1.10, 95% CI 0.65–1.84). At 6 months, there was a statistically significant advantage to dabigatran for all bleeding [16.1% dabigatran v. 21.1% warfarin, (HR 0.71, 95% CI 0.59–0.85)], but no advantage when this was restricted to non-major bleeding (1.6 v. 1.9% respectively, HR 0.82 95% CI 0.45–1.48). Relevant to this review, 3 of the 1265 patients receiving warfarin developed intracranial bleeding, whereas no patients receiving dabigatran suffered an intracranial hemorrhage. While the mechanism of action of Rivaroxaban, Apixaban, and Edoxaban are similar and clinicians can reasonably directly compare much of the data for these agents (pharmacokinetics aside), dabigatran should be considered a different class of agent. This agent is similar in practice to edoxaban in that it is not approved as initial therapy during the first 5 days of treatment, instead needing to be paired with a parenteral bridge. There is extremely limited data in support of its use in cancer patients particularly with CNS disease.

### Direct oral anticoagulants in CNS malignancy and CNS metastatic disease

Data in support of the use of DOAC’s in patients with CNS tumors is emerging but is still quite sparse. However, data in malignancy as a whole has been largely favorable. Carney et al. conducted a retrospective review investigating the rate of intracranial hemorrhage in 172 patients with primary and metastatic disease to brain who were treated with anticoagulation (LMWH v. DOAC) [30]. 67 patients had primary brain tumors, and 20 such patients were treated with DOAC, the other 47 were treated with LMWH. Primary brain tumor patients receiving DOAC had no ICH (0%). The rate of ICH and was significantly higher in those receiving LMWH (36.8%). Likewise, the use of a DOAC did not increase rates of ICH in patients with brain metastases.

The SELECT-D trial represents another important data set contributing to the acceptance of DOAC’s in cancer patients [31]. This trial investigated the use of rivaroxaban compared to LMWH for VTE in active cancer patients. Results were similar to the *Hokusai* trials, but unfortunately, only 3 of the 406 (1.4%) patients randomized had primary brain tumors. The study did report that 58% of patients had metastatic disease, but the percentage of those with intracranial metastases was not specified. The ADAM-VTE trial had a similar design and investigated the safety of apixaban use of in patients with active malignancy. Again, apixaban performed favorably with no major safety surprises [32]. A total of 8 patients had primary brain tumors. The CARAVAGGIO trial is a similar and large prospective trial and represents, in this author’s opinion, a definitive answer to the question in regards to the appropriateness of DOAC’s in most cancer patients. This study was reported in late 2020 and randomized 1170 patients between apixaban and LMWH (dalteparin) [33••]. Apixaban was noninferior to LMWH with no increased risk of recurrent thrombosis, and no increased risk of bleeding. There was arguably a trend towards a reduced rate of recurrent thrombosis for the apixaban arm. Unfortunately, primary brain tumor patients were excluded from this study.

These data and others contributed to the NCCN endorsing DOAC’s as acceptable in the 2018 guidelines for therapeutic anticoagulation [34]. The NCCN now list the following options as category 2A alternatives for patients with reasons to avoid a parenteral anticoagulant as monotherapy: rivaroxaban 15 mg orally BID for 21 days, followed by 20 mg daily, or apixaban 10 mg orally for 7 days, then 5 mg BID. In addition, LMWH for 5–10 days, followed by edoxaban 60 mg orally (or 30 mg for patients with GFR 30–50 ml/min) is now a category 1 NCCN recommendation in recognition of the large cancer-specific trials mentioned above [34]. A parenteral agent, followed by dabigatran, is also recognized, but “limited to those who refuse or have compelling reasons to avoid long-term LMWH.”

In summary, there exists good data gathered over the last 2 decades which support the use of LMWH in cancer patients, with a few studies suggesting this holds true for CNS tumors. Additionally, the clinician can now draw upon prospective and well-randomized data to justify the use of DOAC’s in cancer patients. This includes patients with primary brain tumors and most brain metastasis with caveat of having quite limited data in this patient population and treatment strategy, but the data that is available is largely favorable.

### An approach to initial treatment of DVT/PE identified in a patient with a primary brain tumor or brain metastasis

How does the practicing clinician integrate this collection of data into daily practice?

- **Step 1. Complete a risk assessment.** First, as reviewed above, clinicians must recognize that the risks of untreated VTE are high. However, absolute contraindications to anticoagulation need to be considered, which include recent or ongoing intracranial hemorrhage, severe thrombocytopenia (we favor dose reduction of LMWH for platelet count < 50,000 and discontinuation for platelet count < 25,000/ul) or very high risk, recent intracranial procedure for which anticoagulation is deemed to be contraindicated by the surgeon. Most brain tumors pose a relative risk of bleeding, but do not represent a strict contraindication. Available data suggest that this is true even in most cases for tumors traditionally associated with a “higher” risk of bleeding. Higher risk scenarios appear to include high-grade glioma with or without prior anti-VEGF therapy and untreated metastatic tumors (e.g. pre-radiation). Specific histology associated with higher rates of bleeding include high-grade glioma, choriocarcinoma, malignant pituitary adenoma; for metastatic disease, thyroid carcinoma, melanoma, renal cell carcinoma, and hepatocellular carcinoma are at elevated risk [35]. Again, when encountering the particularly uncomfortable decision of a patient with relative contraindications and indications for anticoagulation, we find it helpful to not only consider the risks of treatment, but also weigh the relative strength of the indication. New, acute, proximal and immediately threatening VTE pose the highest risk and represent a strong indication while isolated distal or asymptomatic thrombosis being somewhat less compelling indications for beginning high risk anticoagulation.
- **Step 2. Choose an agent for initial therapy.** Renal function should be evaluated to clarify if one agent has an advantage. Most data suggest that DOAC’s (rivaroxaban, apixaban, and edoxaban) are equivalent to LMWH, and may have reduced bleeding risk compared to LMWH (discussed above). Additional data will be helpful to confirm that this remains true in patients with CNS tumors. We consider them as reasonable initial therapy, particularly if the patient places high value on avoiding injections.

Rarely, we find it helpful to employ unfractionated heparin (UFH) infusion as initial therapy. This is most often in the admitted and unstable patient with a high-risk thrombosis who is felt to be at exceptionally high risk of bleeding or in need of an imminent procedure. UFH can be helpful due to the short half-life and rapid offset. An additional maneuver that can be considered in the very-high risk patient who is felt to be too high risk to tolerate full-dose anticoagulation, perhaps due to some mild or moderate active bleeding, is to substitute an attenuated, “prophylaxis” dose initially (e.g. enoxaparin 40 mg subcutaneous daily), and gauge clinical stability over a matter of hours. This may offer some initial benefit but low bleeding risk. If well tolerated and bleeding is felt to be stabilized, one can then escalate to “full-dose” anticoagulation the following day.

Inferior Vena Cava (IVC) filters can be used for patients with contraindications to anticoagulation. The enthusiasm for filters has waned in recent years due to increased recognition of their downsides. In CNS disease, IVC filters are most often used in patients with acute thrombosis who are planned for neurosurgery. We recommend that filters be considered for patients only if they are not candidates for anticoagulation for greater than a 72-h window, and have a recent acute thrombosis. The filter should be removed when the contraindication to anticoagulation is resolved.

**Fig. 1:**
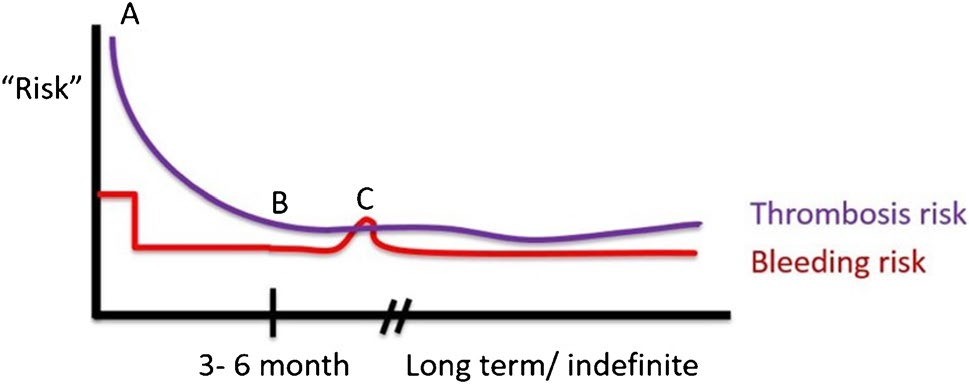
A conceptual framework assisting the assessment of risks and benefits of anticoagulation over time. Time point A. An acute VTE event is associated with a high initial risk. Benefit to anticoagulation is strong. Anticoagulation risk is relatively moderate and may be higher during the initial “loading” phase. B. General risk of the acute thrombosis falls with time and approaches the chronic risk level at 3–6 months. Chronic risk persists, particularly in the setting of malignancy or other risks factors. If the chronic thrombosis risk remains elevated (as in most CNS cancer processes) the benefit to long-term treatment would be expected to exceed the bleeding risk, and there is net benefit to remaining on anticoagulation. Time point C. This risk/benefit balance is not static, and may vary based on the clinical status (e.g., a period of severe thrombocytopenia).

- **Step 3. Consider, and periodically reevaluate, therapy beyond the initial 3–6 months of primary treatment.** The duration of primary treatment for any one acute VTE has been settled at 3 to 6 months. The risk of any one VTE may fall with time as it is treated. Beyond 3–6 months, the thrombosis can be reasonably assumed to be either resolved or converted to a “chronic” thrombus, which poses significantly lower risk of embolism or extension. Beyond 6 months, the benefit of anticoagulation becomes effectively that of secondary prophylaxis, and thus the indication weakens but does not remain fully static (Fig. 1). The patient should have the course of anticoagulant periodically reviewed to better understand the risk and benefit of ongoing treatment. We often consider if there are future procedures planned, review tolerance of anticoagulant to date and also discuss the patient’s ultimate prognosis and goals in the clinic. Owing to the rather high risk of recurrent VTE associated with CNS malignancy, most patients would still be expected to benefit from long-term anticoagulation, at least while the malignancy remains active. There may be periods where holding becomes appropriate (Fig. 1, Time C). For patients who pass a favorable risk/benefit evaluation, we favor indefinite therapy over finite but extended therapy. Thrombophilia testing rarely influences this decision as the risk profile of a prior thrombosis and active high-thrombotic risk malignancy largely drives the risk profile of a recurrent event to a level that warrants long-term anticoagulation, but can be considered for select patients, particularly those with family history.

There are a few other decision points to consider in regards to long-term anticoagulation particularly in the patient who is treated with DOAC’s. Both apixaban and rivaroxaban have been studied at a lower-dose (2.5 mg twice-daily and 10 mg daily, respectively) when entering the secondary prevention phase of treatment [36, 37]. The lower dose of these agents appears to be effective at reducing the risks of thrombosis and may to reduce the risk of bleeding in a dose-dependent manner. For these reasons, for the patient who has too high of a risk to consider indefinite full-dose therapy and who is beyond the acute phase of treatment but has persistent active malignancy or other risk factors, we suggest reducing the dose and continue anticoagulation as tolerated. Finally, the clinician is encouraged to periodically reevaluate the risks and benefits of ongoing treatment, as this balance may not remain static based on the patients clinical course.
